# Modulation the alternative splicing of *GLA* (IVS4+919G>A) in Fabry disease

**DOI:** 10.1371/journal.pone.0175929

**Published:** 2017-04-21

**Authors:** Wen-Hsin Chang, Dau-Ming Niu, Chi-Yu Lu, Shyr-Yi Lin, Ta-Chih Liu, Jan-Gowth Chang

**Affiliations:** 1 Department of Primary Care Medicine, Taipei Medical University Hospital, Taipei, Taiwan; 2 Institute of Clinical Medicine, National Yang-Ming University, Taipei, Taiwan; 3 Department of Pediatrics, Taipei Veterans General Hospital, Taipei, Taiwan; 4 Department of Biochemistry, College of Medicine, Kaohsiung Medical University, Kaohsiung, Taiwan; 5 Research Center for Environmental Medicine, Kaohsiung Medical University, Kaohsiung, Taiwan; 6 Department of General Medicine, School of Medicine, College of Medicine, Taipei Medical University, Taipei, Taiwan; 7 Graduate Institute of Clinical Medicine, College of Medicine, Kaohsiung Medical University, Kaohsiung, Taiwan; 8 Division of Hematology and Oncology, Department of Internal Medicine, Kaohsiung Medical University Hospital, Kaohsiung Medical University, Kaohsiung, Taiwan; 9 Epigenome Research Center, China Medical University Hospital, Taichung, Taiwan; 10 Department of Laboratory Medicine, China Medical University Hospital, Taichung, Taiwan; 11 School of Medicine, China Medical University, Taichung, Taiwan; 12 Department of Bioinformatics and Medical Engineering, Asia University, Taichung, Taiwan; National Cheng Kung University, TAIWAN

## Abstract

While a base substitution in intron 4 of *GLA* (IVS4+919G>A) that causes aberrant alternative splicing resulting in Fabry disease has been reported, its molecular mechanism remains unclear. Here we reported that upon IVS4+919G>A transversion, H3K36me3 was enriched across the alternatively spliced region. PSIP1, an adapter of H3K36me3, together with Hsp70 and NONO were recruited and formed a complex with SF2/ASF and SRp20, which further promoted *GLA* splicing. Amiloride, a splicing regulator in cancer cells, could reverse aberrant histone modification patterns and disrupt the association of splicing complex with *GLA*. It could also reverse aberrant *GLA* splicing in a PP1-dependant manner. Our findings revealed the alternative splicing mechanism of *GLA* (IVS4+919G>A), and a potential treatment for this specific genetic type of Fabry disease by amiloride in the future.

## Introduction

Fabry disease (FD) is an X-linked lysosomal disorder caused by a deficiency of α galactosidase A (GLA), due to mutations in the *GLA* gene at Xq22. The enzymatic defect leads to the accumulation of globotriaosylceramide (Gb3) and related glycosphingolipids throughout the body, causing multisystem disease [1]. Cardiac involvement has been described in FD patients with high prevalence and is one of the major causes of reduced life expectancy [2, 3]. Among the genotype mutations of the *GLA* gene, the intronic mutation at nucleotide 9331 (IVS4+919G>A) is reported to be a cardiac variant Fabry mutation [4–6]. This intronic mutation induces an alternative splicing event in intron 4, which results in an insertion of 57-nt between the exon 4 and 5 of the *GLA* transcript, generating a premature stop codon. The alternatively spliced transcript with 57 nt insertion is rarely expressed in most normal human tissues, but it is predominantly expressed in Fabry disease patients with the IVS4+919G>A mutation. Although the alternatively spliced transcript is reported to be responsible for the reduced enzyme activity causing Fabry disease, the mechanism of *GLA* splicing is unclear.

Alternative splicing, a process that joins different 5’ and 3’ splice sites of an RNA transcript sequence, plays a major role in protein diversity. Splicing of pre-mRNA has been known to be regulated by the spliceosome and approximately 200 additional proteins [7]. The spliceosome recognizes the *cis* sequence elements that define the exon-intron boundaries (the 5’ and 3’splice sites), and catalyzes the splicing reaction. Additional *cis*-acting elements, known as exonic and intronic splicing enhancers or silencers (ESE, ESS, ISE, and ISS), also play a role in the regulation of splicing. *Trans*-acting factors, such as serine/argine-rich (SR) family proteins and heterogeneous nuclear ribonucleoproteins (hnRNPs), can bind to *cis*-acting elements and interact with the spliceosomal complex, thereby controlling the splicing outcomes [8, 9]. In addition, pre-mRNA splicing is initiated co-transcriptionally and is regulated by transcriptional elongation rate. Slow elongation rates facilitate the recognition of weak splice site resulting in exon inclusion, whereas rapid elongation rates lead to exon exclusion [10]. Recent studies have revealed that chromatin structure and histone modifications also play a role in the regulation of alternative splicing [11, 12]. H3K4me3 is reported to enhance the recruitment of spliceosomal components to Interferon regulatory factor 1 (IRF1) and thus facilitates pre-mRNA splicing [13]. H3K36me3 enrichment has been linked to marking exons [14]. Therefore, regulation of alternative splicing is an extensive process and is determined by a combination of chromatin signatures, transcriptional elongation rates, RNA regulatory elements and splicing factors. In this study, we tried to uncover the regulatory mechanism of alternative splicing of *GLA* (IVS4+919G>A) in Fabry disease from chromatin signatures to splicing machinery.

## Results

### Alternative splicing of *GLA* (IVS4+919G>A)

**Fig 1:**
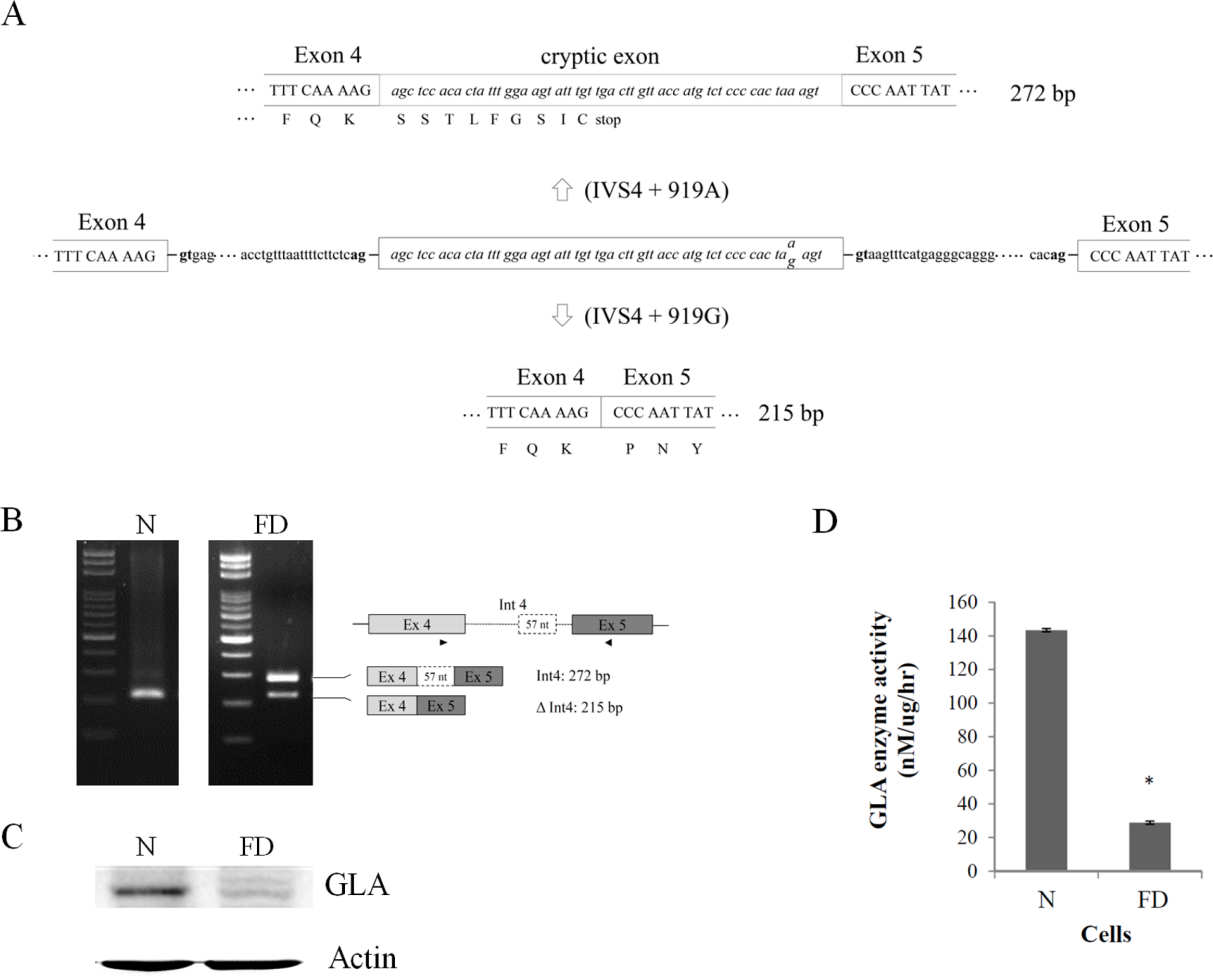
Alternative splicing of *GLA* (IVS4 + 919G>A). (A) Schematic representation of *GLA*. Uppercase letters indicate the exonic sequences, whereas lowercase letters indicate the intronic sequences. The encoded amino acids are depicted in single-letter code. The invariant AG and GT dinucleotides (the 3’ and 5’ splice sites) are shown in boldface type. The alternatively spliced 57 nucleotide sequence is enclosed in the box with italics letters. (B) Messenger RNA was extracted and detected by RT-PCR for alternative splicing of *GLA* (IVS4 + 919G>A). The splicing variants and their expected PCR products using the primers indicated by arrowheads are illustrated on the right column. (C) Aliquots containing 20 μg of whole cell lysates was subjected to SDS-PAGE followed by immunoblot analysis using an anti-GLA antibody. Actin was shown as internal standard. (D) The result of enzyme activity assay from lymphoid cell lines of health person and FD patient. Data were presented as the mean ± standard deviation from three independent experiments. Asterisk represents significant difference (*p*-value < 0.05). N, normal cells; FD, Fabry disease cells.

The genetic organization and splicing pattern of *GLA* were shown in Fig 1A. In order to realize the mechanism of one base transversion leading to the cryptic exon creation, Epstein-Barr virus-transformed lymphoblast cell lines from Fabry disease (FD) patient and health person were established. RT-PCR analysis confirmed that the alternatively spliced intron 4 (the cryptic exon) was weakly expressed in normal cells while it became the dominant product in FD cells (Fig 1B). Western-blot analysis further demonstrated a reduced level of GLA protein in FD cells (Fig 1C), because Int4 inclusion introduced a translation stop codon. Enzyme assay also showed the GLA enzyme activity was decreased in FD cells (Fig 1D).

### Histone modifications on the alternatively spliced region of *GLA*

**Fig 2:**
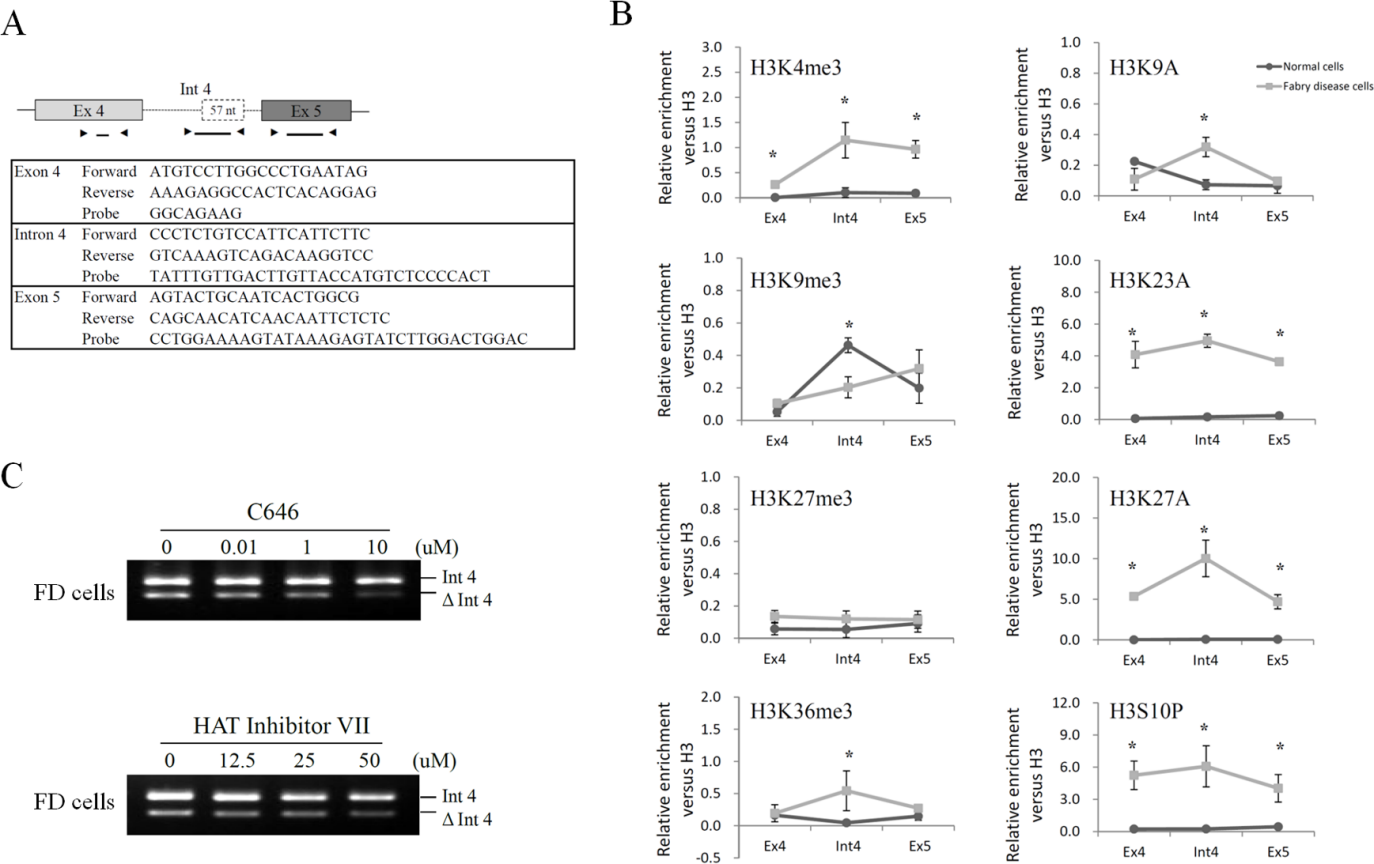
Alterations of histone modifications in FD cells. (A) Schematic representation of position and sequence of primer/probe sets used for real-time PCR. (B) ChIP assays were performed with antibodies to the indicated histone modifications across the alternatively spliced region (exon 4-intron 4-exon 5) of *GLA* in normal cells and FD cells. Results were expressed as a fraction of histone H3 after normalization to input values and presented as a mean values ± standard deviation from at least three independent experiments. Asterisk represents significant difference (*p*-value < 0.05). (C) Fabry disease cells were treated with two different histone acetyltransferase (HAT) inhibitors, C646 and HAT inhibitor VII, for 24 hours. The effects of histone acetylation on alternative splicing of *GLA* were detected by RT-PCR.

To investigate the correlation between histone modifications and alternative splicing, chromatin immunoprecipitation (ChIP) assays were performed using antibodies against a set of histone modifications in FD cells and normal cells. The relative enrichment of each histone modification on *GLA* was quantified by real-time PCR using primer and probe sets targeting exon 4, intron 4 (cryptic exon), and exon 5. Schematic representation of position and sequence of primer/probe sets for real-time PCR are illustrated in Fig 2A. H3K4me3, H3K36me3 and H3S10P were enriched in the cryptic exon in FD cells compared to normal cells, while H3K9me3 was decreased. No significant change of H3K27me3 was found in the cryptic exon between these two cells (Fig 2B). These findings are consistent with earlier reports that H3K36me3 is enriched in exons while H3K9me3 is enriched in introns [15]. Acetylation of H3 at the lysine 9, 23 and 27 was notably high in the cryptic exon with highest increase at the lysine 27 in FD cells. However, inhibition of histone acetyltransferase (HAT) activity by inhibitors C646 and HAT inhibitor VII revealed no significant changes in intron 4 inclusion (Fig 2C) in FD cells. Because histone acetylation has been correlated with transcriptional activation and alternative splicing changes [16–22], we suggested that histone acetylation on *GLA* intron 4 might be involved in transcriptional activation rather than pre-mRNA splicing regulation. Further studies are needed to elucidate which modification on histone H3 is involved in the regulation of *GLA* alternative splicing.

### DNA associated proteins on the cryptic exon area in intron 4 of *GLA*

**Fig 3:**
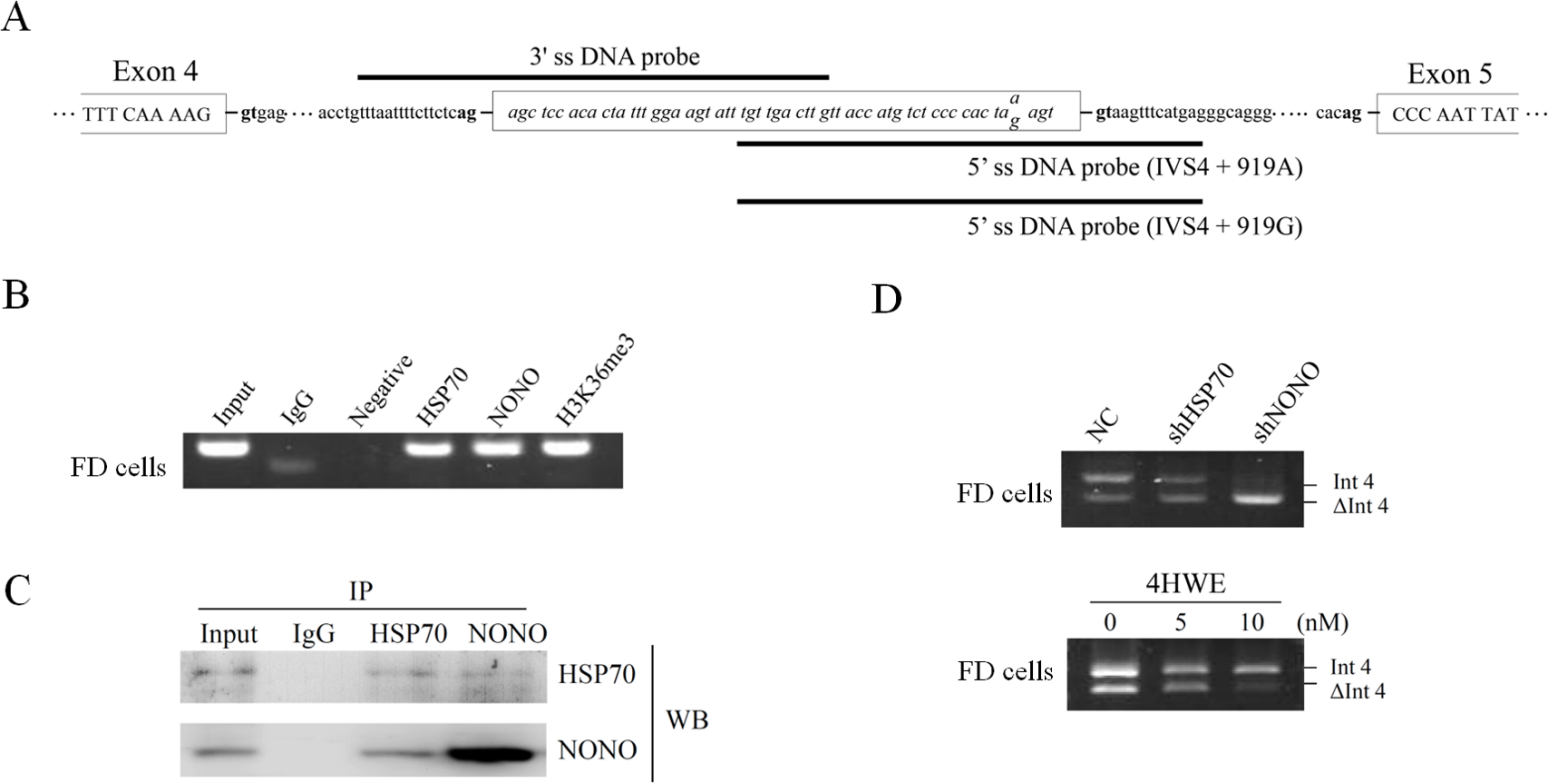
Effects of proteins associated with the cryptic exon area in Int4 of *GLA*. (A) Schematic illustration of *GLA* with positions of the biotin-labelled DNA probes for pull-down assays. (B) ChIP analysis on the cryptic exon area in Int4 of *GLA* was performed using antibodies against HSP70, NONO, and H3K36me3 with IgG as a control. (C) Co-immunoprecipitation results using anti- HSP70 or anti-NONO antibody for immunoprecipitation and analyzing by western blotting. Nonimmune IgG was used as negative control. (D) Fabry disease cells were infected with lentiviruses expressing shRNAs targeting *HSP70* or *NONO*, or treated with 4β-hydroxywithanolide E (4HWE). Messenger RNA was extracted after 48 hours infection or 24 hours 4HWE treatment followed by RT-PCR analysis. WB, western blot; NC: negative control.

Histone modifications can affect pre-mRNA splicing by directly recruiting an adaptor protein, which in turn recruits splicing factors to the nascent RNA. Thus, proteins associated with the DNA template might play a role in the regulation of *GLA* splicing. To reveal whether proteins associated with the DNA template played a role in the regulation of *GLA* (IVS4+919G>A) splicing, streptavidin beads were used to pull down biotin-labelled DNA probes and its associated proteins. Three separate biotin-labelled DNA probes were synthesized and the sequences were shown in Fig 3A. The pull down results showed that PC4 and SFRS1-interacting protein (PSIP1), an adapter of H3K36me3, could bind to the biotin-labelled 3’ splice site DNA probe (S1 Table). Heat shock protein 70 (Hsp70) and non-POU domain-containing octamer-binding protein (NONO) were found to be specifically associated with the DNA probe containing the mutant sequence (IVS4+919A). DNA CHIP analysis further confirmed that Hsp70 and NONO bound to the alternatively spliced region in FD cells (Fig 3B). Moreover, Hsp70 and NONO were demonstrated to be associated with each other in co-immunoprecipitation assay in FD cells (Fig 3C). To explore the role of HSP70 and NONO in *GLA* splicing, we employed an shRNA approach to reduce the expression of HSP70 or NONO in FD cells. Knockdown efficiency was confirmed by western blot analysis (S1 Fig). Knockdown of Hsp70 and NONO reduced the cryptic exon inclusion of *GLA* pre-mRNA in FD cells. Instead, with the use of Golden Berry-Derived 4β-hydroxywithanolide E (4HWE) to enhance the expression of Hsp70 [23] enhanced the cryptic exon inclusion (Fig 3D). These results indicated that DNA associated proteins, which specifically interacted with the alternatively spliced region containing the mutant sequence, played an important role in the regulation of *GLA* (IVS4+919G>A) splicing.

### RNA-associated proteins on the cryptic exon of *GLA* transcripts

**Fig 4:**
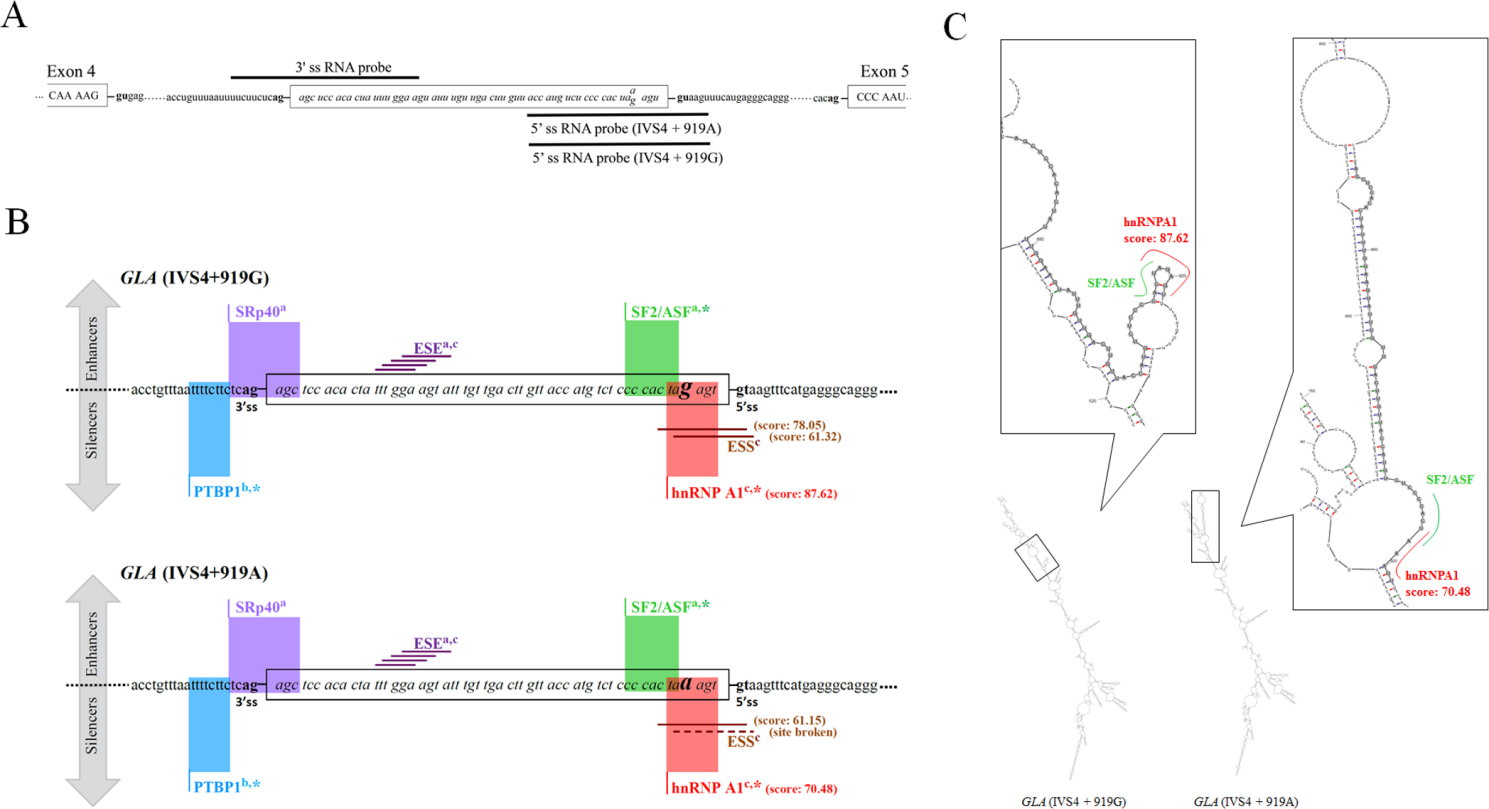
Schematic representation of *GLA* transcripts. (A) Schematic illustration of *GLA* with positions of the biotin-labeled RNA probes for pull-down assays. (B) Putative regulatory motifs and binding sites for the splicing factors as determined by ESEfinder^a^, Spliceaid2^b^, Human Splicing Finder ^c^, and our pull-down experiments*. ESS motifs and hnRNPA1 binding motifs were predicted to be disturbed upon IVS4 + 919G>A transversion. (C) Analyses of the RNA folding of *GLA* (IVS4 + 919G/A). The alternatively spliced 57 nucleotide sequence is highlighted in gray and the boxes indicate the alterations in RNA folding.

**Fig 5:**
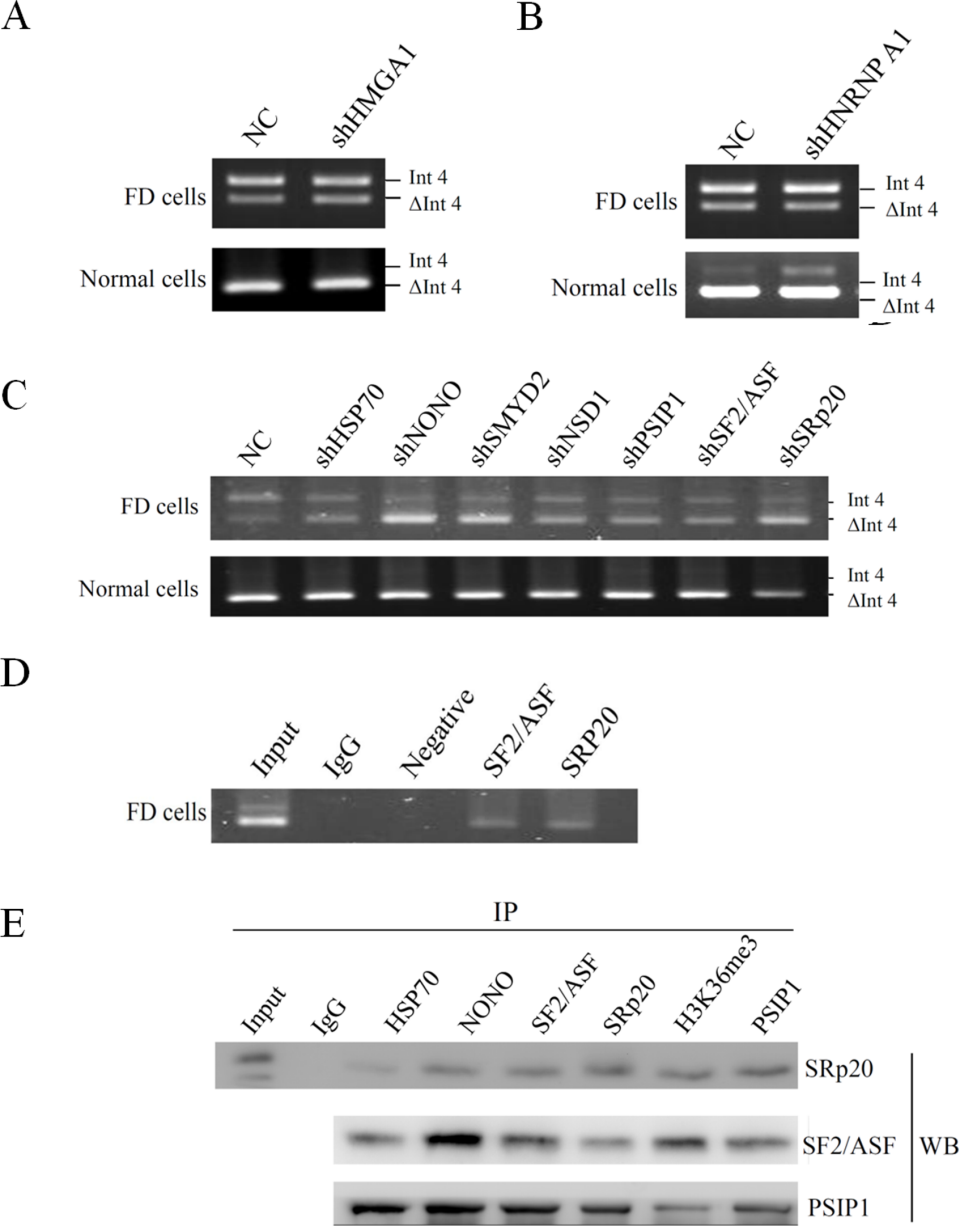
Effects of RNA-associated proteins on the cryptic exon of *GLA* transcripts. (A-C) Virus-mediated shRNA knockdown of various genes as indicated. SMYD2 and NSD1 are histone methyltransferases which preferentially methylates Lys-36 of histone H3. (D) RNA-chromatin immunoprecipitations (RNA-ChIP) analysis on the cryptic exon area in Int4 of *GLA* was performed using antibodies against SF2/ASF and SRp20 with IgG as a control. (E) Co-immunoprecipitation results using the indicated antibodies for immunoprecipitation and analyzing by western blotting. FD cells, Fabry disease cells; NC: negative control; WB, western blot; SMYD2, SET and MYND domain containing 2; NSD1, nuclear receptor binding SET domain protein 1.

To discover which splicing factors are involved in the regulation of *GLA* (IVS4+919G>A) alternative splicing, a biotin pull-down assay was performed. Three separate biotin-labelled RNA probe were synthesized and the sequence were shown in Fig 4A. The pull-down results showed that polypyrimidine tract binding proteins (PTBP1) was associated with the 3’ss RNA probe (S2 Table). Bioinformatics analysis further confirmed that there is a PTBP1 binding motif upstream the 3’ss of the cryptic exon (Fig 4B). With the use of biotin-labelled RNA probes containing the wild type sequence (IVS4+919G) or the mutant sequence (IVS4+919A), we found many hnRNPs, including hnRNP A1, could bind to both of them. However, many splicing inducing proteins, including SF2/ASF and SRp20, and some components of the spliceosome were found to interact only with the RNA probe containing the mutant sequence (IVS4+919A) (S2 Table). HMGA1, instead, was found to bind specifically to the RNA probe containing the wild type sequence (IVS4+919G) without any effect on *GLA* (IVS4+919G>A) splicing (Fig 5A).

Bioinformatics analysis of the alternatively spliced sequences to predict potential regulatory elements in the cryptic exon revealed that no significant changes in ESE motifs, whereas ESS motifs would be disrupted upon IVS4+919G>A transversion as determined by ESEfinder [24, 25] and Human Splicing Finder (HSF) [26, 27] (Fig 4B). Prediction analysis of splicing factor binding sites indicated that the SF2/ASF and hnRNP A1 binding motif was located in the 3’ end of the cryptic exon. The prediction of SpliceAid2 and HSF program further indicated that IVS4+919G>A transversion affected the hnRNP A1 motif, reducing the score from 87.62% to 70.48%. RNA structure analyses of *GLA* using MFOLD [28] program also showed that the IVS4+919G>A transversion was followed by structural changes (Fig 4C).

Base on the bioinformatics software prediction and our pull-down assay results, we further assessed the effects of hnRNP A1 and SR proteins on *GLA* splicing. Knockdown of hnRNP A1 resulted in a mild increase in cryptic exon inclusion in normal cells, demonstrating an inhibitory effect of hnRNP A1 on *GLA* (IVS4+919G) splicing in normal cells (Fig 5B). Instead, knockdown of SF2/ASF and SRp20 reduced cryptic exon inclusion in FD cells. PSIP1, which was found to bind to the 3’ss RNA probe, was reported to bridge pre-mRNA through interactions between H3K36me3, and SF2/ASF and SRp20 [12]. Thus, the effects of H3K36me3 histone mark and PSIP1 on the regulation of *GLA* splicing were evaluated. Knockdown of H3K36 histone methyltransferases and PSIP1 resulted in the decrease of the cryptic exon inclusion in FD cells (Fig 5C). Knockdown efficiency of various target genes was shown in S2 Fig. RNA CHIP analysis confirmed that SF2/ASF and SRp20 associated to the alternatively spliced region (Fig 5D). Coimmunoprecipitation analysis demonstrated that PSIP1 was associated with H3K36me3, SF2/ASF, and SRp20 in FD cells. Hsp70 and NONO were also found to interact with each factor within the H3K36me3/PSIP1/(SF2/ASF and SRp20) complex (Fig 5D). These results indicated that SF2/ASF and SRp20, together with Hsp70, NONO, PSIP1, and H3K36me3, modulated the alternative splicing of *GLA*. Taken together, we suggested that hnRNP A1 played an inhibitory role in *GLA* splicing in normal cells. Upon IVS4+919G>A transversion, the hnRNPA1-dependent splicing silencer motif was disrupted, resulting in an increased recognition of the alternative splice site by SF2/ASF and SRp20.

### Effects of amiloride on the regulation of *GLA* (IVS4+919G>A) splicing

**Fig 6:**
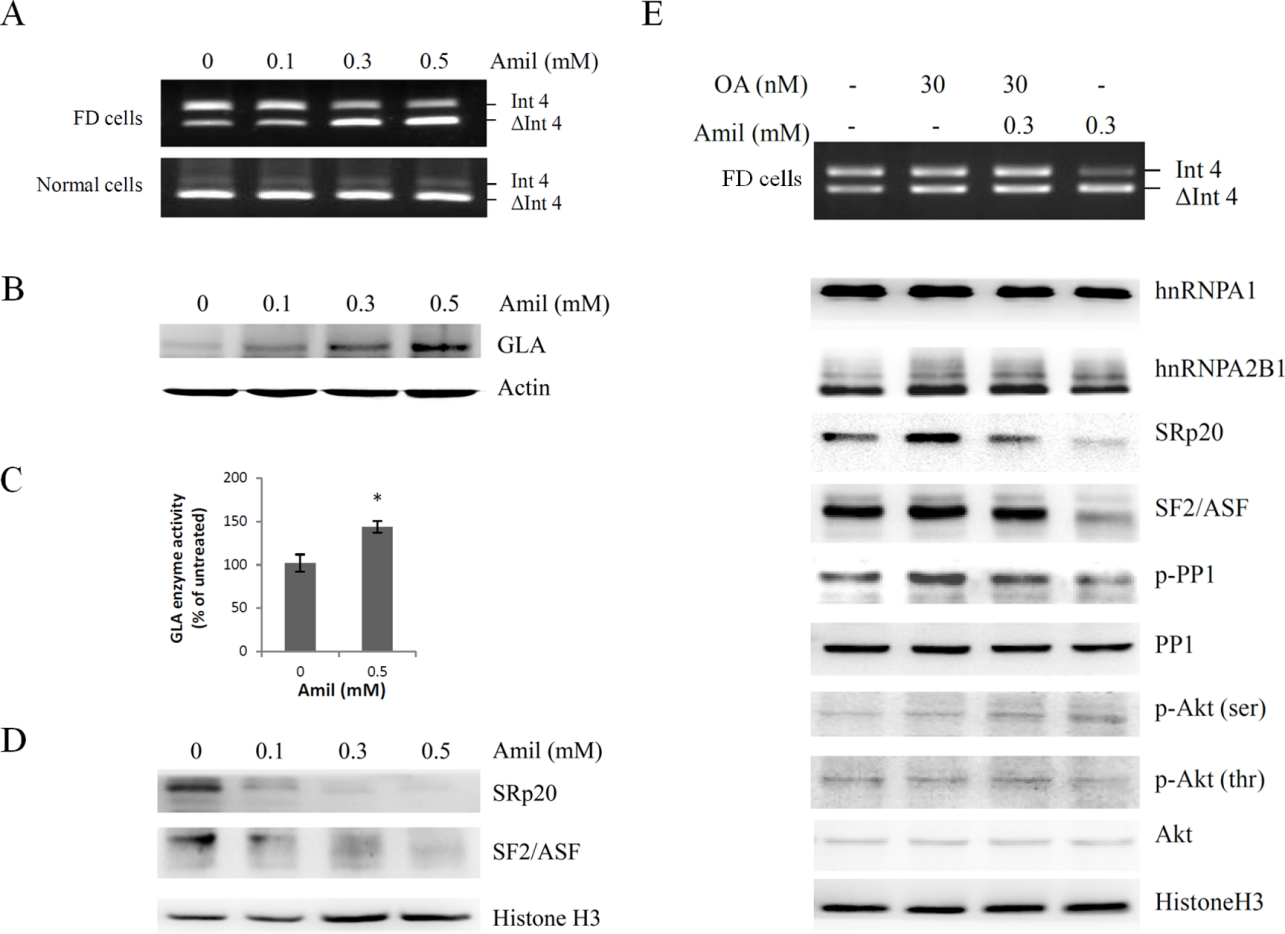
Effects of amiloride on the regulation of *GLA* (IVS4 + 919G>A) splicing. Cells were treated with different concentrations of amiloride for 24 hours and then harvested for RT-PCR analysis (A), or Western blot analysis (B,D). Actin and Histone H3 were used as internal standards. (C) The result of enzyme activity assay from FD cells after the treatment with or without amiloride for 24 hours. Data were presented as the mean ± standard deviation from three independent experiments. Asterisk represents significant difference (*p*-value < 0.05). (E) RT-PCR and Western blot results from cells pretreated with (+) or without (-) okadaic acid and then exposed to amiloride for 24 hours. FD cells, Fabry disease cells; Amil, amiloride; OA, okadaic acid.

Amiloride had the ability to modulate pre-mRNA alternative splicing in different cancer cell lines [29, 30]. We, therefore, speculated whether it could also regulate the alternative splicing of *GLA* (IVS4+919G>A) in FD cells. RT-PCR and western-blot analysis demonstrated that amiloride induced the cryptic exon exclusion of *GLA* (Fig 6A), resulting in increased GLA protein expression (Fig 6B), and enzyme activity (Fig 6C) in FD cells. NONO rather than Hsp70 was disassociated from the biotin-labelled IVS4+919A DNA probe in amiloride-treated FD cells (S3 Table), indicating that NONO played a more important role in amiloride-regulated *GLA* splicing. In addition, splicing factors specifically interacted with IVS4+919A RNA probe were all disrupted by amiloride treatment (S4 Table).

Western blot analysis further revealed that amiloride down-regulated the expression and phosphorylation levels of SF2/ASF and SRp20 in a dose-dependent manner (Fig 6D), but it failed to influence the expression of hnRNP A1 and hnRNP A2B1 (Fig 6E) in FD cells. Since SR proteins are known to be the substrates for Akt kinase and PP1 phosphatase, the decreased level of phosphorylated SF2/ASF and SRp20 may result from either inhibition of Akt kinase activity that catalyzes their phosphorylation or the activation of PP1 phosphatase activity that removes the phosphate moieties from SR proteins, or both. Western blot results showed that amiloride only activated PP1 by the dephosphorylation of Thr^320^, but did not inhibit Akt activity (Fig 6E). With the use of okadaic acid to inhibit PP1 phosphatase activity [29, 31] prior to amiloride treatment, we found it could relieve the effects of amiloride on *GLA* (IVS4+919G>A) splicing, and the phosphorylation levels of SF2/ASF and SRp20. The phosphorylation level of hnRNPs, however, was not affected (Fig 6E). These results indicated that PP1 in part mediated the effects of amiloride on the alternative splicing of *GLA* in FB cells through the dephosphorylation of SR proteins. Taken together, amiloride disrupted the association of splicing complex in the cryptic exon region of *GLA* (IVS4+919G>A), and mediated the alternative splicing in a PP1-dependant manner.

### Effects of amiloride on histone modifications in the cryptic exon area of *GLA*

**Fig 7:**
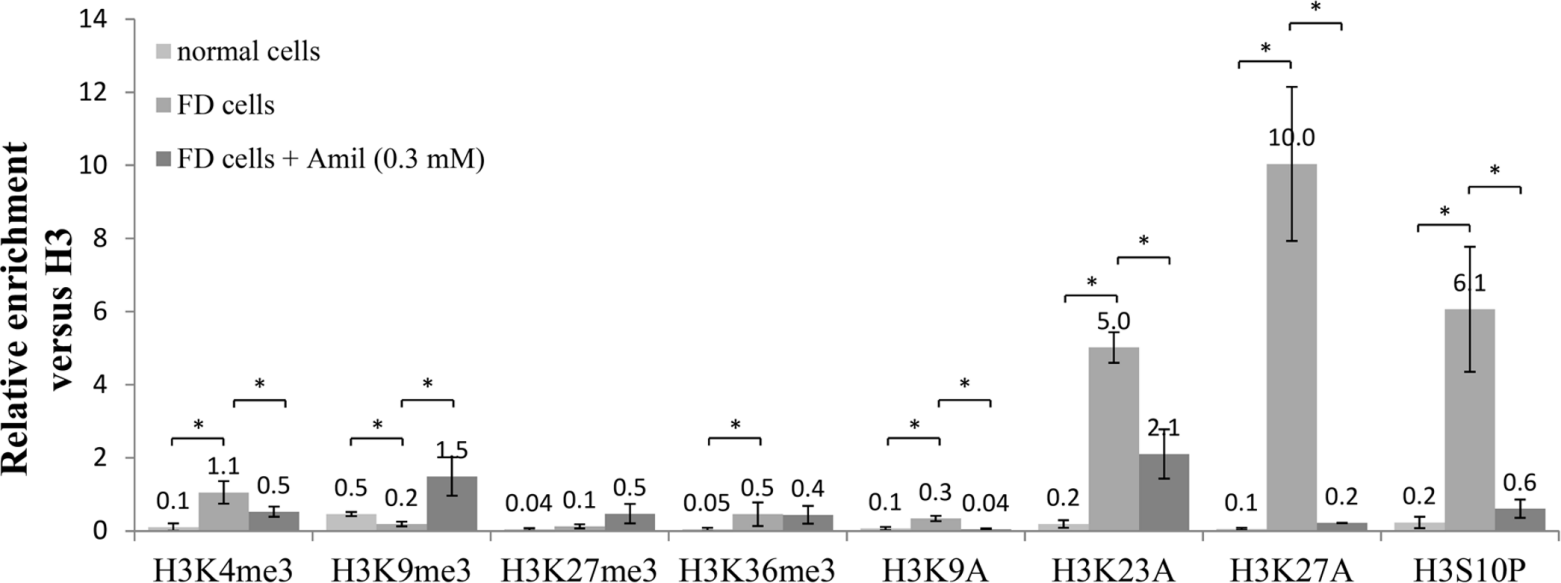
Alterations in histone modification patterns after amiloride treatment. ChIP assays were performed with antibodies to the indicated histone modifications on the cryptic exon area in Int4 of *GLA* in normal cells or in FD cells treated with or without amiloride. Results were expressed as a fraction of histone H3 after normalization to input values and presented as a mean values ± standard deviation from three independent experiments. Asterisk represents significant difference (*p*-value < 0.05). FD cells, Fabry disease cells; Amil, amiloride.

To explore the effects of amiloride on histone modifications in the cryptic exon region, CHIP assays were performed. Although amiloride had no significant effects on H3K27me3 and H3K36me3, it reversed aberrantly elevated levels of H3K4me3, H3S10P and H3 acetylation, and aberrantly reduced levels H3K9me3 in FD cells (Fig 7). These data indicated that amiloride had a global effect on histone modifications and could reverse most of the aberrant modification patterns in FD cells.

## Discussion

The splicing mechanism of the IVS4+919G>A transversion in Fabry disease, which leads to a cryptic exon creation, is unclear. In this study, we clarified its mechanism from chromatin signatures to splicing machinery.

Histone modifications are differentially distributed with respect to intron-exon boundaries, and this differential marking contributes to exon recognition and alternative splicing regulation [32, 33]. For example, H3K9me3 has been found to be enriched in introns and be associated with multiple exon exclusion of CD44 [34]. H3K36me3 has been reported to be more enriched in exons than in introns and be involved in pre-mRNA splicing [27]. Here, we showed that the enriched levels of histone modification were shifted from H3K9me3 to H3K36me3 on the cryptic exon area in Int4 of *GLA* (IVS4+919G>A). We also demonstrated that H3K36me3, PSIP1, SF2/ASF and SRp20 associated with each other and played a role in regulating *GLA* (IVS4 + 919G>A) splicing. Therefore, upon IVS4+919G>A transversion, the enrichment of H3K36me3 may contribute to exon definition and recognition by PSIP1, which in turn recruits it splicing factors and results in the cryptic exon inclusion of *GLA*.

From DNA associated proteins analysis, we found Hsp70 and NONO could specifically recognize the alternatively spliced region containing the mutant sequence (IVS4+919A). Hsp70, well-known as cytosolic chaperone, has been demonstrated to play a role in repairing heat-disrupted splicing and in inhibiting nuclear translocation of the splicing kinases [35, 36]. It has also been reported that Hsp70 acts as a DNA-binding transcriptional co-activator, facilitates transcriptional activation, assists the RNA polymerase II assembly, and modulates the polymerase activity [37–40]. Recently, Hsp70 has been shown to be involved in down-regulation of transcriptional elongation and decrease of nucleosome turnover [41]. Thus, the effects of Hsp70 on *GLA* splicing might through slowing down RNA polymerase II elongation rate to provide enough time for the splicing factors to recognize the weak splicing site, resulting in the cryptic exon inclusion. Further studies are required to assess this hypothesis. NONO has been reported to bind to the polymerase II CTD and RNA transcript simultaneously [42]. It has also been reported to interact directly with the 5’SS within large complexes containing the U1 and U2 snRNPs and RNA polymerase II (RNAPII0) during the coupled process of transcription and splicing. Consistent with these studies, we found NONO could interact near the 5’SS of the cryptic exon in *GLA*. Moreover, we found NONO was associated with Hsp70, SF2/ASF, and SRp20, suggesting its splicing effect might through mediating interactions between transcriptions and splicing machineries.

Lai et al. have described that there are cryptic donor and accepter splice sites, which are not normally activated, in the cryptic exon of normal individuals. Ishii et al. have proposed that *GLA* (IVS4+919G>A) enrichs the A/C predominance for the sequence, acting as an exonic splicing enhancer (ESE) and leading to the inclusion of the cryptic exon. Instead, Palhais et al. have described that *GLA* (IVS4+919G) harbors an hnRNP A1-binding exonic splicing silencer (ESS) that prevents the cryptic exon inclusion. From our results, we revealed that hnRNP A1 played an inhibitory role in *GLA* splicing in normal cells. Upon IVS4+919G>A transversion, an hnRNPA1-dependent splicing silencer motif was disrupted, resulting in an increased recognition of the alternative splice site by SR proteins, including SF2/ASF and SRp20, which further recruits the spliceosome to the cryptic exon, leading to the inclusion of 57-nucleotide of intron 4.

Amiloride is potentially a good agent for cancer therapy by modulating the alternative splicing of various cancer genes [29]. By modulating the alternative splicing of *GLA*, amiloride may play a role in Fabry disease treatment. Consistent with previous study, amiloride regulated the alternative splicing of *GLA* through a PP1-mediated splicing mechanism. In addition, amiloride induced NONO disassociated from the alternatively spliced region. PP1 has been shown to mediate the splicing effects of NONO through regulating its phosphorylation status. Hyperphosphorylated NONO participates in pre-mRNA alternative splicing, whereas dephosphorylated NONO participates in constitutive pre-mRNA splicing [43]. Therefore, PP1 might partially mediate the effects of amiloride on the alternative splicing of *GLA* through the dephosphorylation of NONO.

**Fig 8:**
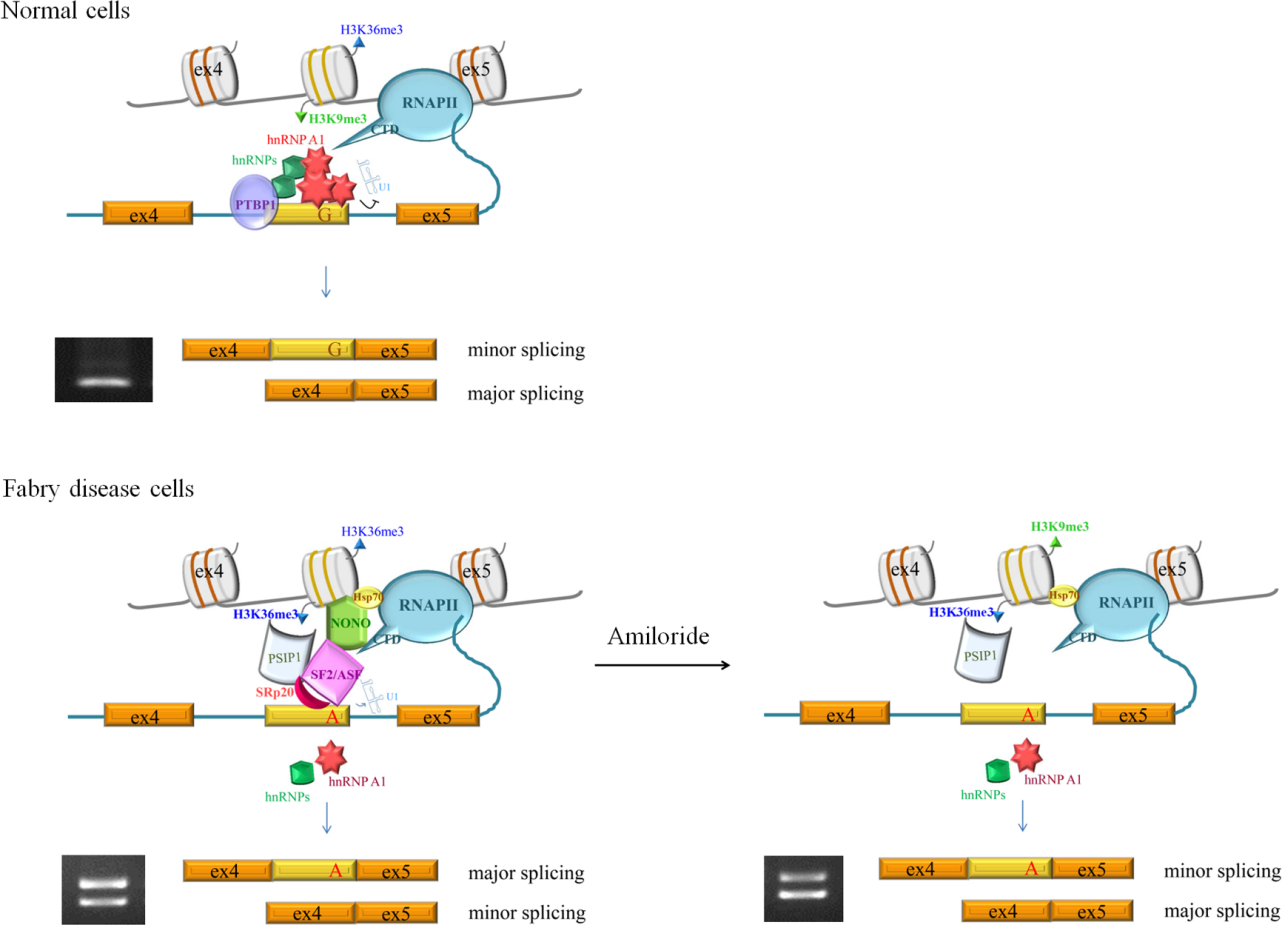
A hypothetical model for the alternative splicing of *GLA* (IVS4 + 919G>A).

In conclusion, we discover that hnRNP A1 plays an inhibitory role in the cryptic exon of *GLA* splicing in normal cells. Upon IVS4+919G>A transversion, the expression of H3K36me3 is enriched on the cryptic exon area in Int4 of *GLA*. PSIP1, an adapter of H3K36me3, together with Hsp70 and NONO are recruited and forms a complex together with the splicing factor, SF2/ASF and SRp20. Besides, this transversion diminishes the splicing silencer motif recognized by hnRNP A1, resulting in an increased recognition of the alternative splice site by SR proteins. Hsp70, NONO, and SR proteins work together to facilitate the recognition of alternative splice site by splicing machinery, resulting in the inclusion of the cryptic exon (Fig 8). We also elucidate the effect of amiloride to modulate *GLA* (IVS4+919G>A) splicing through a PP1 dependent manner, and suggest its role in the treatment of the specific genetic type of Fabry disease. These significant findings reveal the alternative splicing mechanism of *GLA* (IVS4+919G>A), and a potential treatment for this specific genetic type of Fabry disease.

## Materials and methods

### Antibodies

Antibodies were purchased from the following companies: anti-GLA from Santa Cruz; anti-PP1, Akt, phospho-PP1 at Thr^320^, phospho-Akt at Ser^473^, phospho-Akt at Thr^308^ from Cell Signaling Technology; anti-hnRNP A1 from Sigma; anti-hnRNP A2B1 from Acris; anti-SRp20 and SF2/ASF from Thermo Scientific; anti-H3K4me3, anti-H3K9me3, anti-H3K27me3, anti-H3K36me3, anti-H3K9me3, anti-H3K9A, anti-H3K9A, anti-H3K23A, anti-H3K27A, anti-H3S10P, anti-histone H3, anti-actin, and anti-HSP70 from Abcam; anti-p54nrb/NONO from Affinity Bioreagents; anti-PSIP1 from Bethyl Laboratories.

### Ethics statement

The study cohort included adult patients (aged 20 years and older) with Fabry disease diagnosed at Taipei Veterans General Hospital from 2015 to 2017. Epstein-Barr virus-transformed lymphoblast cell lines from FD patient with the IVS4+919G→A mutation and health person were obtained in accordance with an Institutional Review Board-approved protocol at the Taipei Veterans General Hospital (TVGHIRB-2015-04-010C) and informed written consent was obtained from each participant in accordance with the ethical guidelines of the Declaration of Helsinki.

### Cell culture

The Epstein-Barr virus-transformed lymphoblast cells were grown in RPMI-1640 medium supplemented with 2 mM L-glutamine, 10% fetal bovine serum, 100 U/ml penicillin and 100 ug/ml streptomycin at 37°C in a humidified atmosphere containing 5% CO_2_. Amiloride was purchased from Sigma-Aldrich and was dissolved in DMSO (Sigma-Aldrich, St. Louis, MO) to make 500 mM stock solutions. 4β-Hydroxywithanolide E was provided by Professor Y.C., Wu, and was dissolved in DMSO to make 10 mM stock solutions. C646 and HAT inhibitor VII were purchased from Sigma-Aldrich and Millipore. Okadaic acid was obtained from Sigma-Aldrich. C646, HAT inhibitor VII, and okadaic acid were dissolved in DMSO to make 10 mM stock solutions. Serial dilutions were made in DMSO to obtain final dilutions for cellular assays.

### GLA enzyme activity assay

The GLA enzyme activity was measured according to the method described by Desnick et al [44]. In brief, cells were mixed with 300 μl of the substrate solution (5 mM 4-methylumbelliferyl-D-galactopyranoside freshly prepared in 117 mM N-acetyl-D-galactosamine/50 mM citric-phosphate buffer, pH 4.6). After incubation at 37°C for 2 hours, 0.2 N glycine-NaOH was added to stop the reaction. Fluorescence intensity was measured with the excitation and emission wavelengths of 365 and 450 μm, respectively.

### RNA extraction and RT-PCR

We extracted mRNA from the cells using mRNA capture kit (Roche, USA), and converted it into cDNA using RevertAid RT Reverse Transcription Kit (Thermo Fisher Scientific) according to the manufacturer's instructions. For analysis of *GLA* alternatively spliced mRNA isoforms, PCR was performed with forward primer 5’- GTCCTTGGCCCTGAATAG -3’ and reverse primer 5’- GTCCAGCAACATCAACAATT -3’. The PCR was performed with a denaturing step at 94°C for 5 minutes, then 35 cycles of 30 seconds at 94°C, 30 seconds at 58°C and 1 minute at 72°C, followed by a final 5 minutes at 72°C. The PCR products were separated on 2.5% agarose gel and the intensity of the PCR products were analyzed by LabWorks Image Acquisition and Analysis Software (UVP BioImaging Systems). DNA gel bands of these RT-PCR products were isolated for sequencing to verify the authenticity of spliced isoforms.

### Protein extracts and western blotting

Cytoplasmic and nuclear fractions of cells were prepared using NE-PER Nuclear and Cytoplasmic Extraction Reagents (Thermo Scientific) according to the manufacturer's protocol. Total cellular proteins were obtained using Pierce™ IP Lysis Buffer (Thermo Scientific). Protein samples were separated by SDS-PAGE and then transferred to polyvinylidene fluoride membranes (Millipore). The membrane was blocked with 5% BSA and then exposed to the appropriate concentrations of primary antibodies at 4°C overnight. Following PBST washes, membranes were incubated in the appropriate horseradish peroxidase-conjugated secondary antibody for detection by chemiluminescence kit (Amersham Life Science). Intensity of the signals was measured using LabWorks software (UVP BioImaging Systems).

### Coimmunoprecipitation

Coimmunoprecipitation was performed with Pierce Crosslink Magnetic IP/Co-IP kits (Thermo Scientific) according to the manufacturer's protocol. Briefly, 5 μg antibody were covalently cross-linked to 25 μl Protein A/G Magnetic Beads. Equal amounts of cell lysates were immunoprecipitated with the antibody-crosslinked beads at room temperature for 2 hours. After washing, the precipitates were dissociated from the antibody-linked beads by a low-pH elution buffer and analyzed by Western blotting.

### Oligonucleotide pull down assay

Ten micrograms of biotinylated DNA or RNA oligonucleotide corresponding to *GLA* were conjugated with 50 μl of streptavidin resin (Pierce) at 4°C for 30 min. 250 μl of Biotin Blocking Solution was added to block available streptavidin site with free biotin. After three times washing with Tris Buffered Saline, a 300 μl reaction mixture containing 50 μl of nuclear extract and 50 μl of streptavidin beads conjugated with biotinylated RNA was incubated under specific conditions (0.5 mM ATP, 20 mM creatine phosphate, 2.4 mM MgCl_2_, and 20 units of RNasin; Promega) at 4°C for 60 min. After extensive washing with NET-2 buffer (50 mM Tris-HCl, pH 7.4, 150 mM NaCl) containing 0.25% (wt/vol) NP-40, resin-bound proteins were eluted by addition of 250 elution buffer, digested by enzyme and then subjects to Nanoscale capillary liquid chromatography tandem MS (LC-MS/MS) analysis.

### Virus production and gene knockdown

The pLKO shRNA vectors were obtained from the National RNAi Core Facility (Institute of Molecular Biology/Genomics Research Center, Academia Sinica, Taiwan). Lentiviral packaging was performed according to the manufacturer's protocol. Briefly, lentiviral construct was transfected into HEK293T cells using pPACKH1 Lentivector Packing Kit and PureFection Transfection Reagent (System Biosciences, SBI). Virus-containing medium was collected at 72 hours post-transfection. Titrated virus-containing media were used for cells infection.

### ChIP and real-time PCR

ChIP assays were performed with ChIP-IT® Express Chromatin Immunoprecipitation Kits (Active Motif) according to the manufacturer's protocol. In brief, cells were grown to 90% confluence in 150-mm dishes. After crosslinking, cells were lysed in ice-cold complete lysis buffer on ice for 30 min. Nuclei were pelleted at 2400 g for 10 minutes at 4°C, and were resuspended in complete shearing buffer. Chromatin was sheared into 100 bp to 1000 bp fragments by sonication. Ten micrograms of total chromatin was incubated overnight at 4°C with 1 μg antibody. After washing, the immune complex was eluted by adding 100 μl of elution buffer. Subsequently, 2 μl of 5 M NaCl is added to reverse the formaldehyde cross-linking at 65°C for 1.5 h. Following incubation with proteinase K, DNA was obtained and analyzes by real-time PCR. The PCR is performed in a final volume of 10 μl using a LightCycler instrument (Roche Diagnostics) according to the manufacturer’s recommendations. Real-time PCR was performed with the following prime/probe pairs:

Forward Primer: CCCTCTGTCCATTCATTCTTC
Reverse Primer: GTCAAAGTCAGACAAGGTCC
Probe: TATTTGTTGACTTGTTACCATGTCTCCCCACT

## Supporting information

S1 Fig Confirmation of knockdown efficiency by western blot. Western blot analysis showed the knockdown efficiency of HSP70 (A) and NONO (B) in FD cells. Histone H3 was used as an internal control.
(TIF)

S2 Fig Knockdown efficiency of shRNAs. Real-time PCR analysis of various target genes knockdown efficiency in FD cells (A) and normal cells (B), respectively. GAPDH gene was used as an internal gene. Data represent the means±S.D. of three independent experiments. *P<0.05, compared with shCon.
(TIF)

S1 Table MALDI-TOF MS results of cellular proteins binding to the biotin-labelled DNA probes. (DOCX)

S2 Table MALDI-TOF MS results of cellular proteins binding to the biotin-labelled RNA probes. (DOCX)

S3 Table Alterations in DNA-associated proteins by the treatment of amiloride. (DOCX)

S4 Table Alterations in RNA-associated proteins by the treatment of amiloride. (DOCX)
