# Supplementary figures and images for: Modulation the alternative splicing of GLA (IVS4+919G>A) in Fabry disease
Source: PLoS One. 2017 Apr 21;12(4):e0175929. doi: 10.1371/journal.pone.0175929 (PMC5400244; doi:10.1371/journal.pone.0175929)

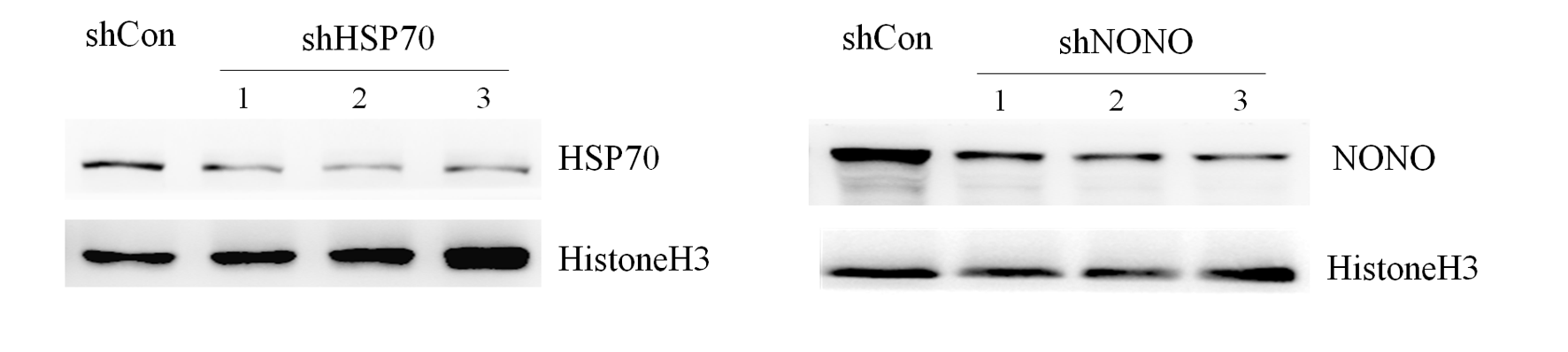

Supplement: S1 Fig — Western blot analysis showed the knockdown efficiency of HSP70 (A) and NONO (B) in FD cells. Histone H3 was used as an internal control. (TIF) [file pone.0175929.s001.tif]

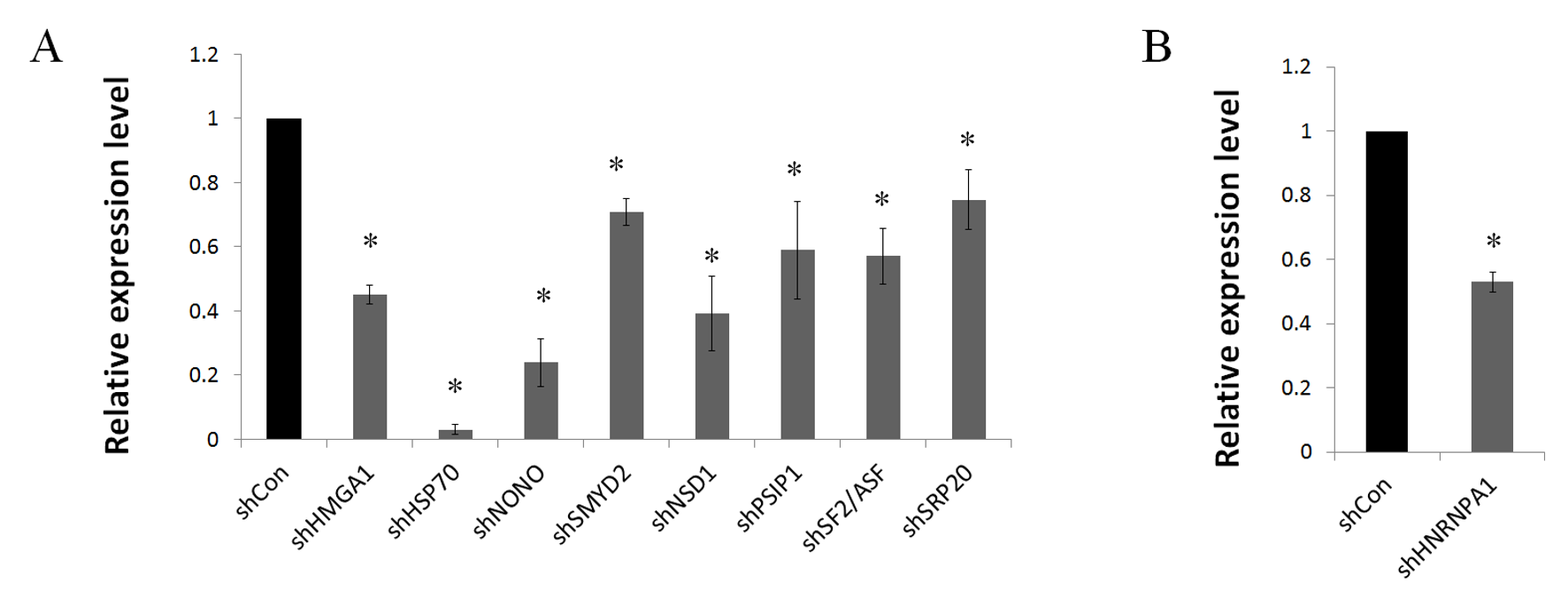

Supplement: S2 Fig — Real-time PCR analysis of various target genes knockdown efficiency in FD cells (A) and normal cells (B), respectively. GAPDH gene was used as an internal gene. Data represent the means±S.D. of three independent experiments. *P<0.05, compared with shCon. (TIF) [file pone.0175929.s002.tif]
