# Supplementary material for: Modulation the alternative splicing of GLA (IVS4+919G>A) in Fabry disease
Source: PLoS One. 2017 Apr 21;12(4):e0175929. doi: 10.1371/journal.pone.0175929 (PMC5400244; doi:10.1371/journal.pone.0175929)
Supplement: S1 Table — (DOCX) [file pone.0175929.s003.docx]

**S1 Table. MALDI-TOF MS results of cellular proteins binding to the biotin-labelled DNA probes**
