# Supplementary material for: Modulation the alternative splicing of GLA (IVS4+919G>A) in Fabry disease
Source: PLoS One. 2017 Apr 21;12(4):e0175929. doi: 10.1371/journal.pone.0175929 (PMC5400244; doi:10.1371/journal.pone.0175929)
Supplement: S3 Table — (DOCX) [file pone.0175929.s005.docx]

| **S3 Table. Alterations in DNA-associated proteins by the treatment of amiloride** | | |
| --- | --- | --- |
|  |  |  |
| **3' ss DNA probe** |  |  |
| protein name | - Amil | + Amil |
| PTBP1 | + | - |
| HMGN1 | + | + |
| PSIP1 | + | + |
|  |  |  |
| **5' ss DNA probe** |  |  |
| **IVS4+919G and IVS4+919A DNA probe** | | |
| protein name | - Amil | + Amil |
| HMGA1 | + | + |
|  |  |  |
| **IVS4+919G DNA probe** |  |  |
| protein name | - Amil | + Amil |
| PTBP1 | + | + |
|  |  |  |
| **IVS4+919A DNA probe** |  |  |
| protein name | - Amil | + Amil |
| NONO | + | - |
| HSP70 | + | + |
|  |  |  |
| - Amil: treated without amiloride; + Amil: treated with amiloride | |  |
