# Supplementary material for: Modulation the alternative splicing of GLA (IVS4+919G>A) in Fabry disease
Source: PLoS One. 2017 Apr 21;12(4):e0175929. doi: 10.1371/journal.pone.0175929 (PMC5400244; doi:10.1371/journal.pone.0175929)
Supplement: S4 Table — (DOCX) [file pone.0175929.s006.docx]

| **S4 Table. Alterations in RNA-associated proteins by the treatment of amiloride** | | |
| --- | --- | --- |
| **3' ss RNA probe** |  |  |
| protein name | - Amil | + Amil |
| HMGN1 | + | + |
| PSIP1 | + | + |
|  |  |  |
| **5' ss RNA probe** |  |  |
| **IVS4+919G and IVS4+919A RNA probe** |  |  |
| protein name | - Amil | + Amil |
| hnRNP A1 | + | + |
| hnRNP A2/B1 | + | + |
| SFPQ | + | + |
| KHSRP | + | + |
|  |  |  |
| **IVS4+919G RNA probe** |  |  |
| protein name | - Amil | + Amil |
| HMGA1 | + | + |
|  |  |  |
| **IVS4+919A RNA probe** |  |  |
| protein name | - Amil | + Amil |
| hnRNP A0, D-like, H, M | + | - |
| SRSF1, 2, 3, 6, 7, 11 | + | - |
| U2AF2 | + | - |
| SNRNPA | + | - |
| SNRNPB, C, E, F, G, Sm D2, Sm D3 | + | - |
| LSm2 , 3, 6, 7 | + | - |
|  |  |  |
| - Amil: treated without amiloride; + Amil: treated with amiloride | | |
